# Efficacy and safety of avacopan in antineutrophil cytoplasmic autoantibody-associated vasculitis: a retrospective cohort study in Japan

**DOI:** 10.1186/s41927-025-00456-4

**Published:** 2025-01-23

**Authors:** Genri Tagami, Makoto Yamaguchi, Hirokazu Sugiyama, Hiroshi Kinashi, Kentaro Imai, Keisuke Kamiya, Takayuki Katsuno, Takahiro Imaizumi, Shogo Banno, Yasuhiko Ito, Takuji Ishimoto

**Affiliations:** 1 Department of Nephrology and Rheumatology, Aichi Medical University, 1-1 Karimata, Yazako, Nagakute, Aichi 480-1195 Japan; 2 Department of Nephrology and Rheumatology, Aichi Medical University Medical Center, Okazaki, Aichi Japan; 3 Data Coordinating Center, Department of Advanced Medicine, Nagoya University Hospital, Nagoya, Japan

**Keywords:** Antineutrophil cytoplasmic autoantibody-associated vasculitis, Avacopan, Efficacy, Remission, Safety

## Abstract

**Background:**

Avacopan, an oral C5a receptor antagonist, demonstrated efficacy as an alternative to glucocorticoid therapy in patients with antineutrophil cytoplasmic antibody (ANCA)-associated vasculitis (AAV) in the phase 3 ADVOCATE trial. However, limited real-world data exist on the outcomes and experiences associated with avacopan use for AAV in Japan.

**Methods:**

We performed a single-centre retrospective analysis and evaluated 21 patients with newly diagnosed or relapsed AAV who received avacopan. The co-primary outcomes were clinical remission at 6 and 12 months.

**Results:**

Among the 21 patients, 20 (95.2%) achieved clinical remission at 6 months, and 19 (90.4%) sustained remission at 12 months. The median time from initiation of immunosuppressive therapy to the start of avacopan was 12 days (interquartile range, 5–26). Adverse events were reported in 10 patients (47.6%), with elevated liver enzyme levels observed in eight patients (38.1%) as the most frequent complication. Avacopan was discontinued in nine patients (42.9%). Despite early discontinuation, these patients achieved comparable rates of clinical remission at 6 months, sustained remission at 12 months, and experienced a reduction in glucocorticoid doses relative to those who continued avacopan.

**Conclusions:**

A high incidence of adverse events, particularly liver enzyme elevation, and frequent early discontinuations of avacopan were observed. Nevertheless, favourable clinical outcomes and reduced glucocorticoid doses were achieved regardless of avacopan discontinuation. Further studies are warranted to validate the optimal use of avacopan in clinical practice.

**Supplementary information:**

The online version contains supplementary material available at 10.1186/s41927-025-00456-4.

## Background

Antineutrophil cytoplasmic antibody (ANCA)-associated vasculitis (AAV) is a group of rare, primary systemic necrotizing vasculitides that affect small blood vessels. The disease encompasses granulomatosis with polyangiitis (GPA) and microscopic polyangiitis (MPA), which account for 80–90% of AAV cases, as well as eosinophilic granulomatosis with polyangiitis [1, 2]. AAV is potentially life-threatening, often presenting with rapidly progressive glomerulonephritis and pulmonary haemorrhage. Prompt initiation of immunosuppressive therapy is crucial to prevent tissue injury and long-term organ damage [2].

The current standard of care for remission induction in AAV involves a combination of cyclophosphamide (CYC) or rituximab (RTX) and glucocorticoids (GCs) [3, 4]. These treatments are highly effective, with remission rates of 80–90% reported in real-world studies [5, 6]. However, prolonged GC exposure is associated with significant adverse events, raising concerns about long-term treatment strategies [7]. To address this, reduced-dose GC regimens from the PEXIVAS [8] and LoVAS trials [9] have been recommended as induction therapies for MPA and GPA [10–12].

Avacopan, an orally administered selective C5a receptor inhibitor, was approved in 2021 for the treatment of AAV in adults. By inhibiting C5a receptors on leukocytes, particularly neutrophils, avacopan reduces inflammation by preventing leukocyte migration and the expression of adhesion molecules [13]. In the phase 3 ADVOCATE trial, which included 331 patients with MPA and GPA (166 treated with avacopan and 165 without), avacopan demonstrated superior sustained remission rates at 52 weeks. Additionally, the cumulative GC use was significantly lower in the avacopan group compared to the prednisone group [13]. Subgroup analysis of the 21 Japanese patients in the trial (11 treated with avacopan and 10 without) yielded similar findings [14].

Despite these promising results, there is limited evidence on the real-world use and outcomes of avacopan for AAV in Japan. To address this gap, we conducted a single-centre retrospective cohort study to evaluate the efficacy and safety of avacopan in Japanese patients with AAV. This study focuses on the relationship between the duration of avacopan use during the induction phase and clinical outcomes.

## Methods

### Study design and patients

This retrospective study included 21 adult patients with newly diagnosed or relapsed AAV who received avacopan as part of their induction treatment between October 2021 and May 2023 at the Nephrology and Rheumatology Centres of Aichi Medical University, Japan. AAV was diagnosed based on the 2012 revised Chapel Hill Consensus Conference criteria and further classified as GPA or MPA using the European Medicines Agency vasculitis algorithm [15]. The study was approved by the Ethics Committee of Aichi Medical University (approval no. 2018-H350) and conducted in compliance with the Declaration of Helsinki. Due to the retrospective nature of the study, the requirement for informed consent was waived.

### Variables

Baseline clinical characteristics were collected at the initiation of immunosuppressive treatment for newly diagnosed or relapsed AAV [16]. These included age, sex, estimated glomerular filtration rate (eGFR, mL/min/1.73 m^2^ = 194 × [serum creatinine]^−1.094^ × [age]^−0.287^ × 0.739 if female) [17], serum C-reactive protein levels, and Birmingham Vasculitis Activity Score (BVAS) 2003 [18]. Details of immunosuppressive therapies were documented, including the use of intravenous CYC, RTX, azathioprine, and methylprednisolone pulse therapy (0.5 or 1.0 g/day for 3 consecutive days); and GC usage, including the daily dose of prednisolone (mg/day) and cumulative doses at 1, 3, 6, and 12 months after initiating induction therapy, was also recorded.

The co-primary outcomes were the proportion of patients achieving clinical remission at 6 months and the proportion of patients maintaining sustained clinical remission at 12 months. Clinical remission was defined as a BVAS of 0 and a daily prednisone dose ≤7.5 mg [19]. Disease relapse was defined as a recurrence of AAV requiring an escalation of immunosuppression at any stage of treatment [20]. Additional outcomes included mortality, progression to end-stage kidney disease (ESKD) requiring dialysis, hospitalization due to infection, and changes in eGFR at 6 and 12 months among patients with kidney involvement.

Safety was assessed by recording treatment-emergent adverse events (TEAEs) associated with avacopan use. TEAEs were defined as adverse events that began or worsened after the initiation of avacopan. Patients were followed until June 1, 2024, and data were censored at death or at the last hospital visit before this date.

### Statistical analyses

Comparisons of clinical characteristics between patients who discontinued avacopan due to TEAEs and those who continued the treatment were conducted using the Wilcoxon rank-sum test or Fisher’s exact test. Statistical significance was defined as *P* < 0.05. All statistical analyses were performed using JMP version 14.0.0 (SAS Institute, Cary, NC) and STATA version 13.0 (StataCorp LP, College Station, TX).

## Results

### Study participants and clinical characteristics

The baseline characteristics of the 21 patients with GPA or MPA initiated avacopan as induction treatment are summarized in Table 1. The median age of the cohort was 77 years (interquartile range [IQR], 66–81), and 11 patients (52.4%) were men. The mean eGFR was 43 mL/min/1.73 m² (IQR, 35–51). Three (14.3%) and 18 (85.7%) patients had GPA and MPA, respectively. Anti- proteinase 3 (PR3), anti-myeloperoxidase (MPO)-ANCA, and ANCA negative were present in two (9.5%), 18 (85.7%), and one (4.8%) patient(s), respectively. Kidney involvement with active urine sediment was present in 15 patients (71.4%), including one patient with eGFR < 15 mL/min/1.73 m². Extrarenal manifestations of vasculitis included 11 patients (52.4%) with lung involvement; three with ear, nose, and throat involvement (14.3%); and six with nervous system involvement (28.6%). All patients had normal liver function tests prior to the initiation of avacopan. Additionally, none had a history of hepatobiliary disease, excessive alcohol consumption, or blood transfusions.

**Table 1:** Baseline characteristics of the patients

	Avacopan (n = 21)
Age, years	77 (66–81)
Male sex, n (%)	11 (52.4%)
Body weight, kg	53 (47–62)
BMI, kg/m^2^	21.2 (19.1–24.4)
eGFR, mL/min/1.73 m^2^	43 (35–51)
Serum albumin, mg/dL	3.2 (2.6–4.0)
CRP level, mg/dL	2.3 (0.5–8.2)
Type of ANCA-associated vasculitis	
GPA	3 (14.3%)
MPA	18 (85.7%)
ANCA-associated vasculitis status	
Newly diagnosed	18 (85.7%)
Relapsed	3 (14.3%)
ANCA positivity	
PR3-ANCA positive	2 (9.5%)
MPO-ANCA positive	18 (85.7%)
ANCA-negative	1 (4.8%)
BVAS	14 (13–17)
Organ involvement	
General	19 (90.4%)
Cutaneous	1 (4.7%)
Ear, nose, and throat	3 (14.3%)
Pulmonary	11 (52.4%)
Diffuse alveolar haemorrhage	0
Interstitial lung diseases	9 (42.9%)
Nodules	2 (9.5%)
Heart	0
Abdominal	0
Nervous system	6 (28.6%)
Kidney	15 (71.4%)
Haematuria	15 (71.4%)
Proteinuria (g/gCr)	0.6 (0.3–1.2)
Rapidly progressive glomerulonephritis	5 (23.8%)

Data are presented as number (%) or median (interquartile range)

*BMI* body mass index, *eGFR* estimated glomerular filtration rate, *CRP* C-reactive protein, *Cr* creatinine, *ANCA* antineutrophil cytoplasmic autoantibody, *GPA* granulomatosis with polyangiitis, *MPA* microscopic polyangiitis, *BVAS* Birmingham Vasculitis Activity Score, *PR3* anti- proteinase 3, *MPO* anti-myeloperoxidase

### Treatment during the observation period

All patients received immunosuppressive therapy (Table 2). Given the retrospective nature of the study, the immunosuppressive therapy protocols were not standardized and were determined by the discretion of the treating physicians. Although the avacopan dose was described to adjust according to the patients’ weight (weight > 55 kg: 30–0–30 mg; weight 40–55 kg: 20–0–20 mg; weight < 40 kg: 10–0–10 mg) [21], in our study, avacopan was prescribed at a dose of 30 mg orally twice daily, initiated at a median of 12 days (IQR 5–26) after the start of remission induction treatment. The remission induction regimens used were: RTX in 16 patients (76.2%), azathioprine in three (14.3%), and methylprednisolone pulse therapy in six (28.6%) patients. When RTX was used as the induction therapy, it was administered intravenously at one or two doses of 375 mg/m^2^/week for 2 weeks. In all cases, sulfamethoxazole/trimethoprim (SMX/TMP) was used for pneumocystis pneumonia prophylaxis. The maintenance treatment regimen was RTX in 15 patients (71.4%), azathioprine in three (14.3%), and mizoribine in one (4.8%). RTX was administered every 6 months as maintenance of remission.

**Table 2:** Immunosuppressive treatment

	**Avacopan (n = 21)**
**Time to start avacopan, days**	12 (5–26)
**Duration of avacopan use, months**	13 (2–13)
**Induction therapy**	
Rituximab	16 (76.2%)
Single dose of rituximab	15 (71.4%)
Two doses of rituximab	1 (4.8%)
Azathioprine	3 (14.3%)
Glucocorticoid monotherapy	2 (9.5%)
Use of methylprednisolone pulse therapy	6 (28.6%)
**Maintenance therapy**	
Rituximab	15 (71.4%)
Azathioprine	3 (14.3%)
Mizoribine	1 (4.8%)
**Prednisone dose**	
Initial dose, mg/day	40 (28–43)
Dose at 1 month, mg/day	8 (5–14)
Dose at 3 months, mg/day	4 (1–5)
Dose at 6 months, mg/day	2 (0–5)
Dose at 12 months, mg/day	0 (0–2)
Cumulative dose at 1 month, g	0.5 (0.3–0.6)
Cumulative dose at 3 months, g	0.6 (0.5–0.8)
Cumulative dose at 6 months, g	0.9 (0.8–1.3)
Cumulative dose at 12 months, g	1.0 (0.9–1.4)
Off prednisone at 6 months	8 (38.1%)
Off prednisone at 12 months	10 (47.6%)

Data are presented as number (%) or median (interquartile range)

The initial median prednisolone dose was 40 mg/day (IQR 28–43). The median doses decreased over time: 8 mg/day (IQR 5–14) at 1 month, 4 mg/day (IQR 1–5) at 3 months, 2 mg/day (IQR 0–5) at 6 months, and 0 mg/day (IQR 0–2) at 12 months. By 6 months, eight patients (38.1%) were able to discontinue prednisolone, and by 12 months, 10 patients (47.6%) had achieved prednisolone withdrawal.

The timeline of individual immunosuppressive treatment, including prednisolone tapering and the timing of avacopan initiation, is illustrated in Supplemental Figure 1.

### Outcomes

The clinical outcomes of the study are summarized in Table 3. Clinical remission was achieved in 20 of 21 patients (95.2%) at 6 months and 19 of 21 patients (90.5%) at 12 months. Among patients with kidney involvement who had follow-up data at 6 or 12 months, the median change in eGFR from baseline was +7 mL/min/1.73 m² (IQR 3–12) at 6 months and +15 mL/min/1.73 m² (IQR 7–21) at 12 months. The baseline eGFR for this subgroup was 43 mL/min/1.73 m² (IQR 35–51). Three patients (14.3%) experienced disease relapse, all of which were minor, presenting only as fever without organ involvement. Hospitalization due to infections occurred in two patients (9.5%), while no cases of ESKD or deaths were reported. There were no significant differences in clinical outcomes observed between 12 months and the final follow-up.

**Table 3:** Outcomes

	Avacopan (n = 21)
**Primary outcome**	
Clinical remission at 6 months	20 (95.2%)
Clinical remission at 12 months	19 (90.5%)
**Secondary outcomes**	
Clinical relapse	3 (14.3%)
Change in eGFR, mL/min/1.73 m^2^ (baseline to 6 months)	+7 (3–12)
Change in eGFR, mL/min/1.73 m^2^ (baseline to 6 months)	+15 (7–21)
ESKD	0
Infection requiring hospitalization	2 (9.5%)
Death	0
BVAS at 6 months	0
BVAS at 12 months	0
Observation period, months	14 (13–18)

Data are presented as number (%) or median (interquartile range)

*eGFR* estimated glomerular filtration rate, *ESKD* end-stage kidney disease, *BVAS* Birmingham Vasculitis Activity Score

### Safety

TEAEs associated with avacopan were observed in 10 patients (47.6%) after a median duration of 1.6 months (IQR 1.0–1.9) of avacopan use (Table 4). The most common TEAEs included elevated liver enzyme levels in eight patients (38.1%) and gastrointestinal symptoms, such as diarrhoea or nausea, in two patients (9.5%). Hepatic events involved aspartate aminotransferase (AST) or alanine aminotransferase (ALT) levels exceeding three times the upper limit of normal, without concurrent elevation in bilirubin levels, and occurred at a median of 1.7 months (IQR 1.5–1.9) after starting avacopan.

**Table 4:** Safety

	Avacopan (n = 21)
**TEAEs associated with avacopan**	10 (47.6%)
Elevated liver enzymes	8 (38.1%)
AST level, U/L (normal range; 13–30)	142 (135–143)
ALT level, U/L (normal range; 30–42)	153 (142–183)
Diarrhoea/Nausea	2 (9.5%)
**Drug discontinuation due to TEAEs associated with avacopan**	9 (42.9%)
Elevated liver enzymes	7 (33.3%)
Diarrhoea/Nausea	2 (9.5%)
**Time to drug discontinuation, months**	1.6 (1.0–1.9)

Data are presented as number (%) or median (interquartile range)

*TEAEs* treatment-emergent adverse events, *AST* aspartate transferase, *ALT* alanine transaminase

In all cases of hepatic events, hepatobiliary imaging showed no abnormalities, and viral hepatitis was excluded based on serological testing (negative HAV-IgM, HBV-DNA, HCV antibody, and cytomegalovirus antigenemia). Avacopan was discontinued in nine patients (42.9%) due to adverse events—seven cases of hepatic injury and two cases of diarrhoea/nausea—at a median of 1.6 months (IQR 1.0–1.9) after starting treatment. Following drug discontinuation, all symptoms resolved, and elevated liver enzyme levels normalized within 2 weeks, despite the continuation of other medications such as SMX/TMP, vitamin D, and anti-gastric acid drugs. In one patient who experienced elevated liver enzymes, the avacopan dose was reduced from 60 to 20 mg/day based on the physician’s judgement. The patient’s weight was 70 kg and no drug interactions required dose adjustment. Although the dose may have been insufficient, this dose adjustment resulted in the normalisation of liver enzyme levels, enabling the continuation of avacopan at the reduced dose. No liver biopsies were performed in this study.

### Comparison between the avacopan discontinuation and continuation groups

To evaluate whether avacopan discontinuation due to TEAEs impacted the treatment regimen and clinical outcomes, comparisons were made between patients who discontinued avacopan (n = 9) and those who continued the treatment (n = 12) (Supplemental Tables 1–3). The analysis revealed that the group discontinuing avacopan was significantly older than the continuation group, with median ages of 79 years (IQR 74–82) versus 70 years (IQR 60–78), respectively (*P* = 0.030) (Supplemental Table 1). However, no significant differences were observed between the two groups regarding immunosuppressive therapy protocols (including induction and maintenance treatments), GC doses throughout the observational period, or clinical outcomes (Supplemental Tables 2 and 3).

## Discussion

This retrospective cohort study found that avacopan, used during the induction treatment phase, achieved high clinical remission rates at 6 months, maintained remission at 12 months, and facilitated reduced GC doses, aligning with previous studies [5, 6, 9]. However, a notable frequency of elevated liver enzyme levels and a high discontinuation rate were observed early in treatment. Interestingly, patients who discontinued avacopan still achieved comparable remission rates at 6 and 12 months with GC dose reduction. The study aimed to explore the relationship between the duration of avacopan use during induction therapy and clinical outcomes in AAV patients.

A phase 3 ADVOCATE trial involving 21 Japanese patients (11 treated with avacopan) demonstrated its efficacy in achieving disease remission with reduced GC use [13]. At 26 weeks, remission rates were comparable between the avacopan and prednisone groups (72% vs. 70%), while sustained remission at 52 weeks was higher in the avacopan group (66% vs. 55%). Subgroup analyses in Japanese patients corroborated these findings [14].

Several observational studies [20, 22–26] (Table 5) have also reported outcomes with avacopan. In a U.S. post-marketing study by Zonozi [20], involving 92 AAV patients, remission rates were 90% at 26 weeks and 84% at 52 weeks, similar to the findings in this study.

**Table 5:** Clinical characteristics and outcomes of AAV patients treated with avacopan in the literature

Author, Country, Year	Number of patients	Age	Organ involvement	Definition of remission	Outcomes
Zonozi, USA, 2024 [20]	92	59 ± 17	• Renal: 71 (77%) • Pulmonary: 48 (52%)	No signs or symptoms of vasculitis activity and ≤5 mg prednisolone	• Remission at 26 w: 61 (90%) • Remission at 52 w: 32 (84%) • Relapse: 3 (3%)
Draibe, Spain, 2024 [22]	29	56 (IQR 46.5–67.5)	• Renal: 24 (79.3%) • Pulmonary: 9 (31.0%)	BVAS 0	• Remission: 25 (86.2%) • Relapse: 4 (13.7%)
Falde, Spain, 2024 [23]	15	66 (IQR 52–72)	• Diffuse alveolar hemorrhage: 15 (100%) • Renal: 9 (60%)	BVAS 0 with complete discontinuation of prednisone	• Remission: 10 (66%) • Relapse: 0 (0%)
Barr, Canada, 2024 [24]	4	59–80	• eGFR < 15 ml/min per 1.73 m^2^: 4 (100%)	No signs or symptoms of vasculitis activity	• Remission: 0 (0%)
Chalkia, UK, 2024 [25]	8	64 (17–80)	• Hypoxic pulmonary hemorrhage: 8 (100%)	Complete resolution of lung symptoms	• Complete resolution; 8 (100%) • Survival and sustained remission: 8 (100%)
Zimmermann, Germany, 2024 [26]	39	64 (IQR 51–72)	• Renal: 33 (85%) • Pulmonary: 20 (51%)	BVAS 0 and ≤7.5 mg prednisolone	• Remission at 6 months; 28 (87.5%) • Sustained remission at 12 months: 21 (91%) • Relapse: 4 (10%)
The present study	21	77 (IQR 66–81)	• Renal: 15 (71.4%) • Pulmonary: 11 (52.4%)	BVAS 0 and ≤7.5 mg prednisolone	• Remission at 6 months; 20 (95.2%) • Remission at 12 months: 19 (90.5%) • Relapse: 3 (14.3%)

*eGFR* estimate glomerular filtration rate, *BVAS* Birmingham Vasculitis Activity Score

However, regarding safety, this study observed a higher incidence of elevated liver enzymes and treatment discontinuation compared to previous studies [13, 20]. For example, the ADVOCATE trial [13] reported liver function test abnormalities in 5.4% of avacopan-treated patients, while Zonozi [20] found this in 4.3% of cases. In contrast, the current study noted a higher frequency of these events, possibly due to the following factors: (1) Genetic predisposition: Drug-induced liver injury (DILI) has been linked to human leukocyte antigen haplotypes, potentially explaining higher DILI rates in Japanese patients [14, 27]. (2) Age-related vulnerability: Older patients, particularly those with hepatotoxicity, may experience slower metabolic processes, leading to toxic metabolite accumulation in liver tissue [14].

Drug interactions with avacopan warrant consideration, particularly the need to adjust doses of CYP3A4-sensitive substrates [27] and avoid grapefruit, grapefruit juice, or related supplements. This study did not assess such interactions, highlighting the need for further investigation. Elevated liver enzyme levels might also stem from the combination of avacopan with SMX/TMP or RTX, both of which have been associated with liver damage despite not being CYP3A4 inhibitors. Further research is essential to determine the mechanism behind liver injury in these scenarios.

Concerning the severity of liver damage associated with avacopan in our study, as in previous studies [13, 20], liver enzyme levels normalized after stopping avacopan; however, several case reports [28, 29] have shown severe liver damage, presenting with vanishing bile duct syndrome. Increasing evidence indicates that the complement system is actively involved in the pathogenesis of various liver disorders, including liver injury, repair, and fibrosis [30], suggesting that blocking the C5a receptor by avacopan leads to liver damage. Therefore, conditions predisposing an individual to the development of severe liver damage due to avacopan.

Notably, early avacopan discontinuation did not impair the achievement or maintenance of remission or GC dose reduction at 6 and 12 months. This may reflect the benefits of initiating avacopan promptly after AAV diagnosis when complement activation is highest, enabling effective disease suppression and rapid GC tapering. Further well-designed studies are needed to refine strategies for optimal avacopan use.

## Study limitations

This study has several limitations. First, as a retrospective study, unmeasured confounding factors could not be adjusted for due to variability in treatment regimens among patients. Additionally, it remains unclear which patients with AAV genuinely require avacopan. Future research should focus on identifying patient subgroups that would benefit most from this therapy. Second, the small sample size limits the generalizability of the findings, highlighting the need for well-designed, large cohort studies. Third, the short observation period was insufficient to assess the long-term efficacy and safety of avacopan in AAV, a condition that often necessitates prolonged treatment. Fourth, the study included only two PR3-ANCA-positive patients, which limits the applicability of findings to the broader AAV population. Fifth, no patient received CYC as an induction agent, and RTX was administered as a single dose in older patients for remission induction. This approach is not standard of care and may be insufficient in many cases. Thus, the results should be interpreted with caution, and further studies are needed to validate these findings.

Sixth, previous studies employed varying definitions of clinical remission based on prednisolone doses, such as prednisolone 0 mg [13], ≤5 mg [20], and ≤7.5 mg, as used in our study [19].

These differences should be considered when comparing outcomes across studies. Lastly, the proportion of patients with low eGFR (<15 mL/min/1.73 m²) was lower than that in other studies [20, 22–24, 26], and no patients in this study experienced pulmonary haemorrhage. These factors suggest that disease severity in this study was milder compared to previous research. As a result, the findings should be interpreted cautiously.

## Conclusions

The use of avacopan during the remission induction phase demonstrated high clinical remission rates at 6 and 12 months while enabling GC dose reduction. However, the frequent occurrence of TEAEs, particularly elevated liver enzyme levels and a high rate of early discontinuation, remains a significant concern. Notably, favourable outcomes and potential steroid reduction were observed even in patients who discontinued avacopan early. Further studies are essential to validate the optimal use of avacopan and address these safety concerns.

## Electronic supplementary material

Below is the link to the electronic supplementary material.

Supplementary Material 1 Supplemental Figure 1. Timeline of individual immunosuppressive treatment, including prednisolone, and the timing of avacopan initiation for all 21 patients (avacopan continuation group (n = 12, patients 1–12) and discontinuation group (n = 9, patients 13–21))

Supplementary Material 2 Supplemental Table 1. Comparison of baseline characteristics between avacopan continuation and discontinuation groups

Supplementary Material 3 Supplemental Table 2. Comparison of immunosuppressive treatment between avacopan continuation and discontinuation groups

Supplementary Material 4 Supplemental Table 3. Comparison of outcomes between avacopan continuation and discontinuation groups

## Data Availability

The data that support the findings of this study are available on request from the corresponding author. The data are not publicly available due to privacy or ethical restrictions.

## Abbreviations

ANCA: Antineutrophil cytoplasmic autoantibody
AAV: ANCA-associated vasculitis
AST: Aspartate transferase
ALT: Alanine transaminase
BVAS: Birmingham Vasculitis Activity Score
BMI: Body mass index
CRP: C-reactive protein
CYC: Cyclophosphamide
DILI: Drug-induced liver injury
eGFR: Estimated glomerular filtration rate
ESKD: End-stage kidney disease
GPA: Granulomatosis with polyangiitis
GC: Glucocorticoid
IQR: Interquartile range
MPA: Microscopic polyangiitis
MPO: Myeloperoxidase
PR3: Proteinase 3
RTX: Rituximab
SMX/TMP: Sulfamethoxazole/trimethoprim
TEAE: Treatment-emergent adverse events
