# Supplementary figures and images for: Efficacy and safety of avacopan in antineutrophil cytoplasmic autoantibody-associated vasculitis: a retrospective cohort study in Japan
Source: BMC Rheumatol. 2025 Jan 23;9:8. doi: 10.1186/s41927-025-00456-4 (PMC11756139; doi:10.1186/s41927-025-00456-4)

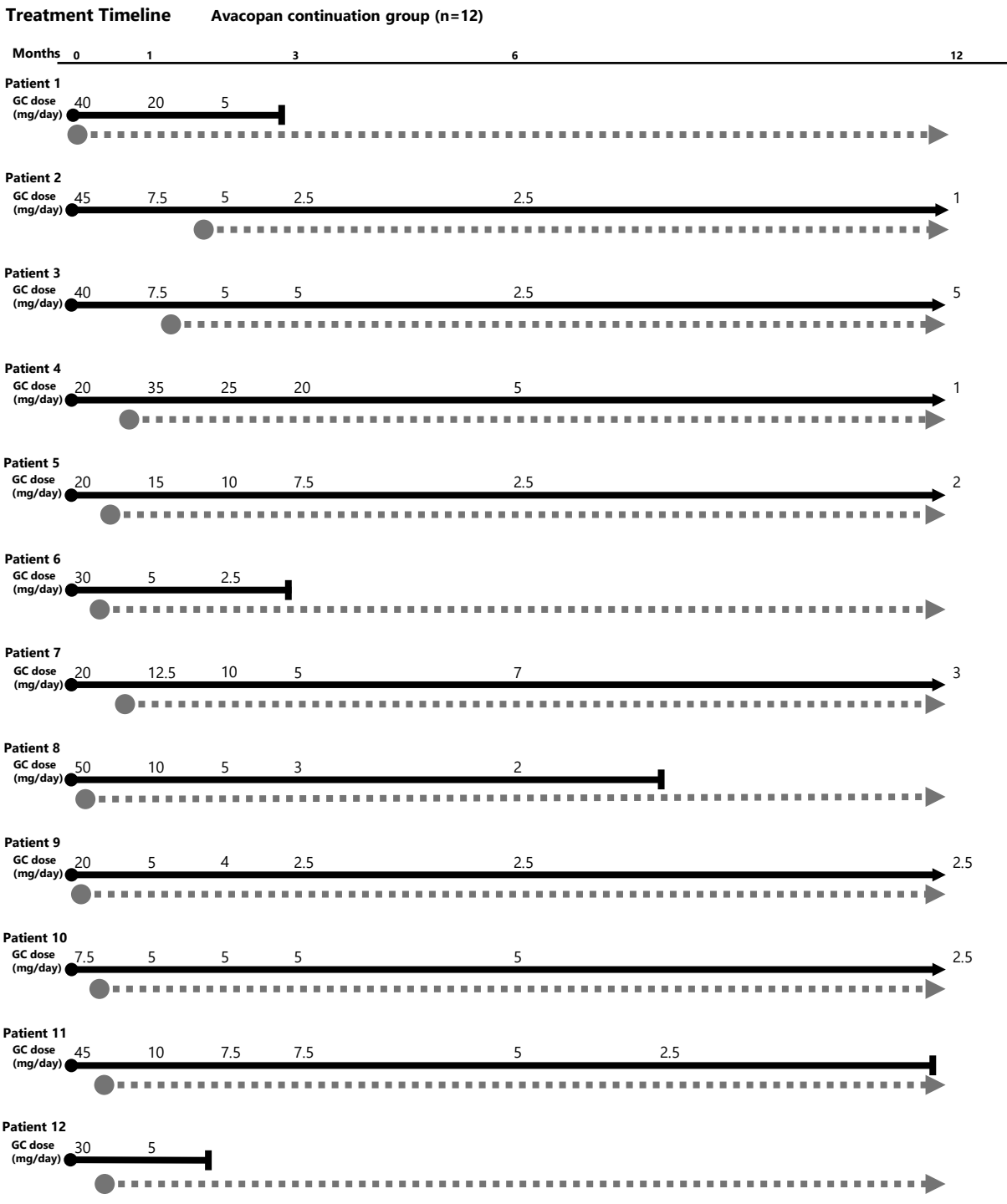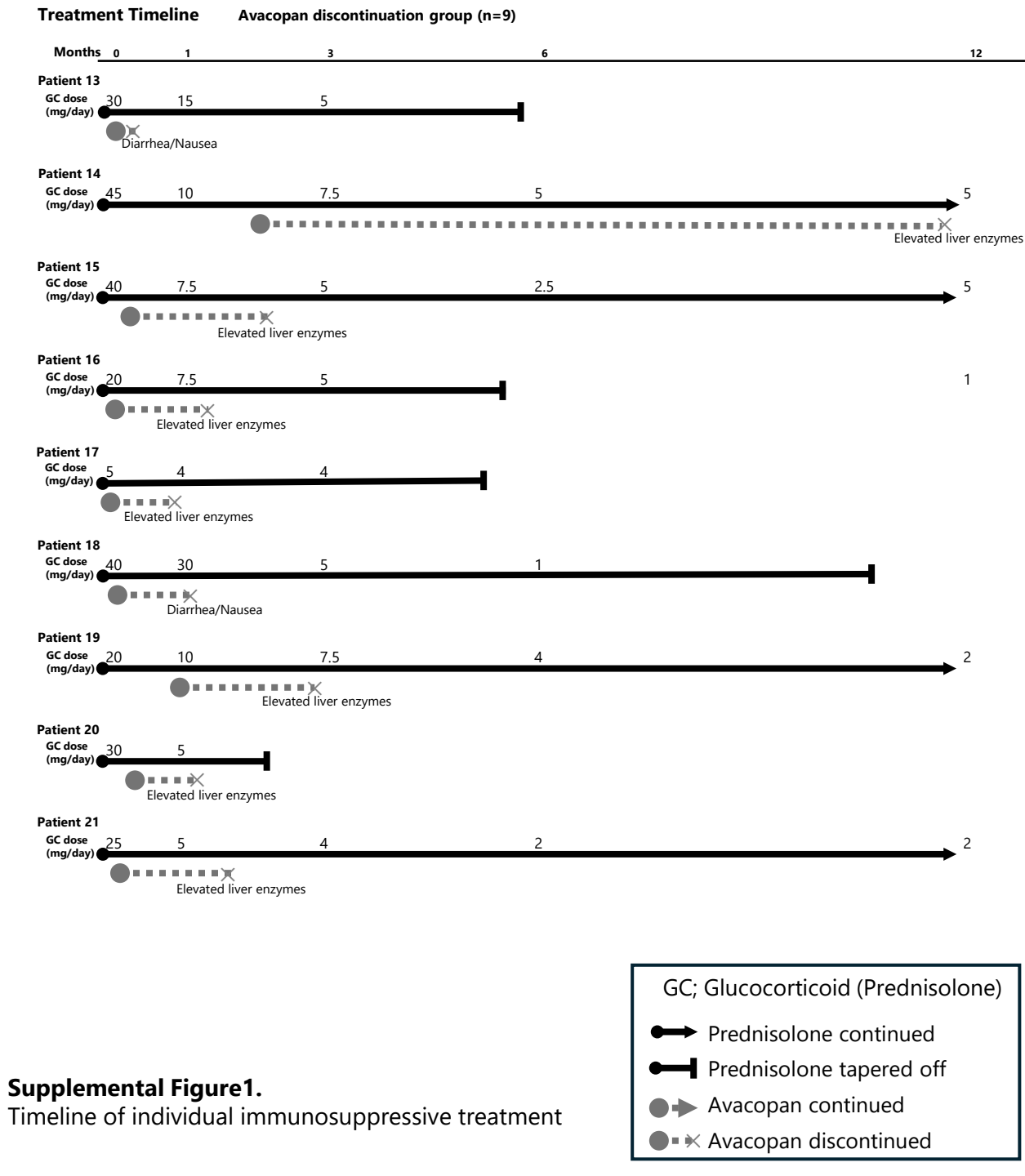

**Supplemental Figure1.**  
Timeline of individual immunosuppressive treatment

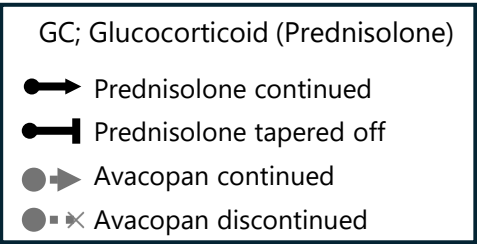

Supplement: Supplementary file 1 — Supplementary Material 1 Supplemental Figure 1. Timeline of individual immunosuppressive treatment, including prednisolone, and the timing of avacopan initiation for all 21 patients (avacopan continuation group (n = 12, patients 1–12) and discontinuation group (n = 9, patients 13–21)) [file 41927_2025_456_MOESM1_ESM.pdf]
