# Supplementary material for: Efficacy and safety of avacopan in antineutrophil cytoplasmic autoantibody-associated vasculitis: a retrospective cohort study in Japan
Source: BMC Rheumatol. 2025 Jan 23;9:8. doi: 10.1186/s41927-025-00456-4 (PMC11756139; doi:10.1186/s41927-025-00456-4)
Supplement: Supplementary file 2 — Supplementary Material 2 Supplemental Table 1. Comparison of baseline characteristics between avacopan continuation and discontinuation groups [file 41927_2025_456_MOESM2_ESM.docx]

**Supplemental Table 1.** **Comparison of baseline characteristics between avacopan continuation and discontinuation groups**

|  | **Duration of avacopan** | |  |
| --- | --- | --- | --- |
|  | **Continuation (n=12)** | **Discontinuation (n=9)** | *P*-value |
| Age, year | 70 (60–78) | 79 (74–82) | 0.030 |
| Male sex | 7 (58.3%) | 4 (44.4%) | 0.528 |
| BMI, kg/m^2^ | 21.7 (18.1–24.6) | 20.8 (19.4–23.9) | 0.831 |
| Body weight, kg | 53 (46–67) | 52 (47–57) | 0.972 |
| eGFR, mL/min/1.73 m^2^ | 43 (30–55) | 41 (38–49) | 1.000 |
| Serum albumin, mg/dL | 3.6 (3.0–4.0) | 2.8 (1.0–4.0) | 0.144 |
| CRP level, mg/dL | 1.7 (0.6–6.0) | 6.1 (0.3–9.6) | 0.522 |
| Type of ANCA-associated vasculitis |  |  | 0.719 |
| GPA | 2 (16.7%) | 1 (11.1%) |  |
| MPA | 10 (83.3%) | 8 (88.9%) |  |
| ANCA-associated vasculitis status |  |  | 0.105 |
| Newly diagnosed | 9 (75.0%) | 10 (100%) |  |
| Relapsed | 3 (25.0%) | 0 |  |
| ANCA positivity |  |  | 0.513 |
| PR3-ANCA positive | 2 (18.2%) | 1 (10.0%) |  |
| MPO-ANCA positive | 9 (81.8%) | 8 (80.0%) |  |
| ANCA-negative | 0 | 1 (10.0%) |  |
| BVAS | 15 (10–16) | 15 (14–16) | 0.423 |
| Organ involvement |  |  |  |
| General | 10 (83.3%) | 9 (100%) | 0.198 |
| Cutaneous | 0 | 1 (11.1%) | 0.237 |
| Ear, nose, and throat | 2 (18.2%) | 1 (11.1%) | 0.423 |
| Pulmonary | 6 (50.0%) | 5 (55.6%) | 0.801 |
| Diffuse alveolar hemorrhage | 0 | 0 |  |
| Interstitial lung diseases | 5 (41.7%) | 4 (44.4%) | 0.907 |
| Nodules | 1 (8.3%) | 1 (11.1%) | 0.830 |
| Heart | 0 | 0 |  |
| Abdominal | 0 | 0 |  |
| Nervous system | 3 (25.0%) | 3 (33.3%) | 0.676 |
| Kidney | 8 (66.7%) | 7 (77.8%) | 0.577 |
| Hematuria | 8 (66.7%) | 7 (77.8%) | 0.577 |
| Proteinuria, g/gCr | 0.7 (0.1–1.1) | 0.2 (0.1–0.7) | 0.159 |
| Rapidly progressive glomerulonephritis | 3 (25.0%) | 2 (22.2%) | 0.882 |

Data are presented as number (%) or median (interquartile range). BMI, body mass index; eGFR, estimated glomerular filtration rate; CRP, C-reactive protein; ANCA, antineutrophil cytoplasmic autoantibody; GPA, granulomatosis with polyangiitis; MPA, microscopic polyangiitis; BVAS, Birmingham Vasculitis Activity Score; ESKD, end-stage kidney disease; Cr, creatinine; BVAS, Birmingham Vasculitis Activity Score; PR3, anti-proteinase 3; MPO, anti-myeloperoxidase.
