# Supplementary material for: Efficacy and safety of avacopan in antineutrophil cytoplasmic autoantibody-associated vasculitis: a retrospective cohort study in Japan
Source: BMC Rheumatol. 2025 Jan 23;9:8. doi: 10.1186/s41927-025-00456-4 (PMC11756139; doi:10.1186/s41927-025-00456-4)
Supplement: Supplementary file 3 — Supplementary Material 3 Supplemental Table 2. Comparison of immunosuppressive treatment between avacopan continuation and discontinuation groups [file 41927_2025_456_MOESM3_ESM.docx]

**Supplemental Table 2**: **Comparison of immunosuppressive treatment between avacopan continuation and discontinuation groups**

|  | **Duration of avacopan** | |  |
| --- | --- | --- | --- |
|  | **Continuation (n=12)** | **Discontinuation (n=9)** | *P*-value |
| **Treatment** |  |  |  |
| **Time to start avacopan, days** | 13 (7–26) | 7 (4–23) | 0.374 |
| **Duration of avacopan use, months** | 13 (13–14) | 1.6 (1.0–1.9) | <0.001 |
| **Induction therapy** |  |  | 0.630 |
| Rituximab | 10 (83.3%) | 6 (66.7%) |  |
| Azathioprine | 1 (8.3%) | 2 (22.2%) |  |
| Use of methylprednisolone pulse therapy | 4 (33.3%) | 2 (22.2%) | 0.577 |
| **Maintenance therapy** |  |  | 0.674 |
| Rituximab | 9 (75.0%) | 6 (66.7%) |  |
| Azathioprine | 2 (16.7%) | 1 (11.1%) |  |
| Mizoribine | 0 | 1 (11.1%) |  |
| **Prednisone dose** |  |  |  |
| Initial dose, mg/day | 40 (26–44) | 30 (25–45) | 0.856 |
| Dose at 1 month, mg/day | 8 (5–14) | 10 (5–12.5) | 0.914 |
| Dose at 3 months, mg/day | 3 (0–5) | 5 (4–6) | 0.261 |
| Dose at 6 months, mg/day | 2 (0–3) | 2 (0–5) | 0.661 |
| Dose at 12 months, mg/day | 1 (0–3) | 0 (0–2) | 0.336 |
| Cumulative dose at 1 month, g | 0.4 (0.3–0.6) | 0.5 (0.3–0.5) | 1.000 |
| Cumulative dose at 3 months, g | 0.6 (0.4–0.8) | 0.6 (0.5–0.8) | 0.915 |
| Cumulative dose at 6 months, g | 0.9 (0.8–1.3) | 1.0 (0.8–1.5) | 0.639 |
| Cumulative dose at 12 months, g | 1.0 (0.9–1.5) | 1.1 (0.9–1.6) | 0.998 |
| Off prednisone at 6 months | 5 (41.7%) | 3 (33.3%) | 0.697 |
| Off prednisone at 12 months | 7 (58.3%) | 3 (33.3%) | 0.256 |

Data are presented as number (%) or median (interquartile range).
