# Supplementary material for: Efficacy and safety of avacopan in antineutrophil cytoplasmic autoantibody-associated vasculitis: a retrospective cohort study in Japan
Source: BMC Rheumatol. 2025 Jan 23;9:8. doi: 10.1186/s41927-025-00456-4 (PMC11756139; doi:10.1186/s41927-025-00456-4)
Supplement: Supplementary file 4 — Supplementary Material 4 Supplemental Table 3. Comparison of outcomes between avacopan continuation and discontinuation groups [file 41927_2025_456_MOESM4_ESM.docx]

**Supplemental Table 3.** **Comparison of outcomes between avacopan continuation and discontinuation groups**

|  | **Duration of avacopan** | |  |
| --- | --- | --- | --- |
|  | **Continuation (n=12)** | **Discontinuation (n=9)** | *P*-value |
| **Primary outcome** |  |  |  |
| Clinical remission at 6 months | 11 (91.7%) | 9 (100%) | 0.375 |
| Clinical remission at 12 months | 11 (91.7%) | 8 (88.9%) | 0.830 |
| **Secondary outcomes** |  |  |  |
| Clinical relapse | 3 (25.0%) | 0 | 0.105 |
| Change in eGFR, mL/min/1.73 m^2^ (baseline to 6 months) | +6 (4–13) | +7 (4–12) | 0.761 |
| Change in eGFR, mL/min/1.73 m^2^ (baseline to 12 months) | +16 (11–22) | +14 (5–18) | 0.494 |
| ESKD | 0 | 0 |  |
| Infection requiring hospitalization | 2 (16.7%) | 0 | 0.198 |
| Death | 0 | 0 |  |
| BVAS at 6 months | 0 | 0 | 0.387 |
| BVAS at 12 months | 0 (0–3) | 0 | 0.115 |
| Observation period (months) | 13 (13–16) | 16 (14–20) | 0.145 |

Data are presented as number (%) or median (interquartile range). eGFR, estimated glomerular filtration rate; BVAS, Birmingham Vasculitis Activity Score; ESKD, end-stage kidney disease
